# A Role of Medial Olivocochlear Reflex as a Protection Mechanism from Noise-Induced Hearing Loss Revealed in Short-Practicing Violinists

**DOI:** 10.1371/journal.pone.0146751

**Published:** 2016-01-08

**Authors:** Sho Otsuka, Minoru Tsuzaki, Junko Sonoda, Satomi Tanaka, Shigeto Furukawa

**Affiliations:** 1 NTT Communication Science Laboratories, NTT Corporation, 3-1 Morinosato Wakamiya, Atsugi, Kanagawa 243-0198, Japan; 2 Kyoto City University of Arts, 13-6 Kutsukake-cho, Oe, Nishikyo-ku, Kyoto 610-1197, Japan; University of Salamanca- Institute for Neuroscience of Castille and Leon and Medical School, SPAIN

## Abstract

Previous studies have indicated that extended exposure to a high level of sound might increase the risk of hearing loss among professional symphony orchestra musicians. One of the major problems associated with musicians’ hearing loss is difficulty in estimating its risk simply on the basis of the physical amount of exposure, i.e. the exposure level and duration. The aim of this study was to examine whether the measurement of the medial olivocochlear reflex (MOCR), which is assumed to protect the cochlear from acoustic damage, could enable us to assess the risk of hearing loss among musicians. To test this, we compared the MOCR strength and the hearing deterioration caused by one-hour instrument practice. The participants in the study were music university students who are majoring in the violin, whose left ear is exposed to intense violin sounds (broadband sounds containing a significant number of high-frequency components) during their regular instrument practice. Audiogram and click-evoked otoacoustic emissions (CEOAEs) were measured before and after a one-hour violin practice. There was a larger exposure to the left ear than to the right ear, and we observed a left-ear specific temporary threshold shift (TTS) after the violin practice. Left-ear CEOAEs decreased proportionally to the TTS. The exposure level, however, could not entirely explain the inter-individual variation in the TTS and the decrease in CEOAE. On the other hand, the MOCR strength could predict the size of the TTS and CEOAE decrease. Our findings imply that, among other factors, the MOCR is a promising measure for assessing the risk of hearing loss among musicians.

## Introduction

Excessive noise exposure can cause temporary hearing deterioration as well as permanent hearing damage to the cochlear, which is known as noise induced hearing loss (NIHL). Previous studies have indicated that professional symphony orchestra musicians are exposed to a high level of sound, comparable to noise in industrial settings (between 79 and 98 dBA [1]), and are at risk of hearing loss [1], [2]. Indeed, some previous studies reported a permanent threshold shift (PTS) among professional symphony orchestra musicians (e.g., [3], [4], [5]).

At present, the risk factors for musicians’ hearing loss have not been sufficiently identified. A considerable number of studies have assessed the risk of NIHL among musicians by sound exposure. The degree of risk varies with environmental factors such as instrument type and position in the orchestra. Nevertheless, the variation of PTS has only partially been explained by the amount of exposure: Some studies reported that groups with a higher exposure level tend to show larger PTSs [3], [6], while other studies failed to demonstrate any association between exposure level and the PTS [7], [8], [9], [10]. It has also been reported that thresholds among musicians cannot be predicted from ISO-1999 [8], [11].

Although the environmental factors involved in musicians’ hearing loss have been intensively investigated, as mentioned above, very little attention has been focused on the inter-individual variation of susceptibility to NIHL in musicians. Previous studies on occupational hearing loss have revealed that the amount of hearing loss depends on the susceptibility to NIHL, which varies substantially between individuals, as well as on the amount of exposure [12]; individuals with ‘tough’ ears are more resistant to acoustic overexposure, whilst individuals with ‘tender’ ears are more vulnerable [13].

To identify factors underling the variability of susceptibility, previous studies have examined various biological factors such as eye color, gender, age, and middle-ear muscle reflex, and environmental factors such as exposure to drugs or chemicals [14]. Among them, the most promising predictor of susceptibility was the strength of the medial olivocochlear reflex (MOCR) [15]. Outer hair cells (OHCs) receive innervations from the medial part of the superior olivary complex (SOC) through MOC bundles. Those MOC fibers are activated by acoustic stimulation and induce an inhibition effect on OHC motility. This suppressive effect has been termed MOCR. There are two kind of MOCR: ipsilateral and contralateral MOCR. The ipsilateral MOCR is double crossing reflex: Ipsilateral acoustic stimulation activates the contralateral MOC neurons via crossed MOC bundles, and these MOC neurons innervate the ipsilateral ear via crossed MOC bundles. In contrast, the contralateral MOCR is single crossing reflex: Contralateral acoustic stimulation activates the ipsilateral MOC neurons via crossed MOC bundles, and these MOC neurons innervate the ipsilateral ear via uncrossed MOC bundles.

Both types of MOCR have been implicated in protecting the ear from acoustic damage, although their usefulness in natural environment is under debate [16]: Several animal studies have demonstrated that electrical or acoustical stimulation of the MOC bundle decreases the amount of temporary threshold shift (TTS) [17], [18] and sectioning of the MOC bundle increases the amount of PTS after noise exposure [19]. An experimental animal study reported that the MOCR strength could predict the size of the PTS induced by loud noise moderately well [15]. Importantly, Maison et al. [15] assessed the MOCR as a suppression of otoacoustic emission (OAE), which is assumed to reflect changes in the OHC function related to the MOCR [20]. This assessment can potentially be applied to humans [21].

Despite extensive studies [22], the evidence for the protective role of MOCR in *humans* is still equivocal. It was only recent that Wolpert et al. [23] reported a significant correlation between MOCR-related OAE suppression and the size of TTS induced by intense white noise. Even under similar laboratory-controlled conditions, however, Collet et al. [24] failed to find a correlation between the TTS and the amount of MOCR-related OAE suppression. Engdahl [25] reported an even “positive” correlation between OAE amplitude change after exposure to intense band noise and the amount of MOCR-related OAE suppression. Under field study conditions, Veuillet et al. [26] found a significant correlation between MOCR strength and the amount of threshold recovery three days after exposure to rifle blasts. However, other field studies—a gun-shot exercise [26], [27], music [28], discotheque [29], and occupational noise [30]—failed to find evidence for a correlation between MOCR strength and the size of the TTS.

We hypothesized that MOCR measurement could be applied to musicians to assess their risk of NIHL, because musicians’ MOCRs are stronger than non-musicians [31], [32] and should also show a large variation, thereby being likely to determine their risk of NIHL. Nevertheless, this possibility has not been explored, despite a considerable number of studies on NIHL among musicians.

To test this hypothesis, we examined whether MOCR strength can predict the hearing deterioration caused by short-duration instrument practice. The temporary hearing deterioration was quantified by measuring audiograms and click-evoked otoacoustic emissions (CEOAEs) before and after a one-hour instrument practice. OAEs can exhibit an observable change before changes in conventional audiometry associated with noise exposure [33] and may describe hearing deterioration more clearly. We chose violinists as participants. Because of the proximity of the violin to the left ear, the left ear is exposed to intense violin sounds (broadband sounds containing a significant number of high-frequency components) during regular instrument practice. Violinists therefore have a higher probability of NIHL with potential interaural differences than other musicians [5], [6], [34].

## Methods

### Participants

Sixteen audiometrically normal college-age violinists participated in the experiment. They were students of Kyoto City University of Arts, majoring in violin. None reported a clinical history of hearing disorders. Three participants (#10, #14, and #15) whose CEOAE signal-to-noise ratio was too low (< 2 dB) were not included in the post-experiment statistical analysis relevant to the OAE and the MOCR data. Their distributions of their age, gender and violin-playing experience are summarized in Table 1. All participants signed a consent form. The experiments were approved by the Ethics Committee of NTT Communication Science Laboratories, and were conducted in accordance with the Declaration of Helsinki.

**Table 1:** Participants’ demographic data. The participants who did not show a measurable OAE are marked with asterisk i.e., #10, #14 and #15.

Participant no.	Gender	Age [years]	Music experience [years]
1	Female	21	15.5
2	Female	22	15
3	Female	20	16
4	Female	22	19
5	Female	21	16
6	Female	22	17
7	Female	20	17
8	Female	22	16
9	Female	19	13
10*	Female	19	15.5
11	Female	20	17
12	Female	20	15
13	Female	19	14
14*	Female	22	19
15*	Male	18	15
16	Female	19	16
		AVE 20.4 (SD 1.3)	AVE 16.0 (SD 1.3)

### Equipment

For CEOAE and MOCR measurement, stimuli were digitally synthesized with sampling rates of 96 kHz and converted to analog signals using an Edirol UA-101 (24 bits). The analog signals were amplified by a headphone buffer and presented through Etymotic Research ER-4s earphones connected to an ER-10B low-noise microphone system. Ear-canal sound pressure was recorded with an Etymotic Research ER-10B low-noise microphone system inserted in each ear. Sounds for eliciting the ipsilateral and contralateral MOCR were delivered by ER-4s earphones. Prior to the measurement, the outputs from the ER-4s were calibrated using a DB2012 accessory (external ear simulator) of a Bruel and Kjaer Type 4257 ear simulator (IEC 711).

Sounds during the practice were recorded with custom-made equipment comprising two microphones (SOUNDMAN OKM-II) and headbands. This equipment allowed us to place two small microphones 1–2 cm away from either ear. Recording of the practice session were made by directing the line output of the microphones to a TASCAM DR-05 linear PCM recorder with a 44.1-kHz sampling rate and 16-bit resolution. Prior to the measurement, the frequency characteristics of the microphones were calibrated with Bruel and Kjaer BK4192 microphones. All measurements and violin practices were conducted in a quiet classroom or conference room at Kyoto City University of Arts.

### Audiogram measurement

Hearing thresholds were measured with an audiometer (RION Type AA-58) and expressed as hearing level (in dB HL). The thresholds were measured at five frequencies (500, 1000, 2000, 4000, and 8000 Hz) with 5-dB intensity resolutions using the audiometer’s search algorithm. The order of the right- and left-ear measurement was randomized for each participant.

### MOCR and CEOAE measurement

We measured both the ipsilateral and contralateral MOCRs as CEOAE suppression induced by noise presented to the ipsilateral and contralateral ear, respectively. The amount of suppression, typically ~ 4 dB, was assumed to be related to the MOCR strength [35].

In the measurement of the contralateral MOCR, a continuous noise was presented to the contralateral (opposite) ear during the CEOAE recording [21]. The noise was band-pass filtered between 100 and 10000 Hz and had a duration of 6 s, including a 10-ms raised-cosine ramp. The noise was presented at 60-dB SPL. The click had a duration of 100μs and was presented at 60-dB peak-equivalent SPL. We chose a low intensity click and elicitor noise to avoid producing additional hearing loss and eliciting the middle-ear reflex. One measurement block was composed of without-noise and with-contralateral-noise conditions. In the without-noise condition, a click alone was presented 50 times at intervals of 30 ms without presenting the elicitor noise. In the with-contralateral-noise condition, the same click was presented 250 times during presentation of the noise to the contralateral ear. In each measurement block, the without-noise condition was always presented before the with-contralateral-noise condition.

In the measurement of the ipsilateral MOCR, we applied the forward suppression paradigm [36], in which click was presented after the elicitor noise with a silent gap. The MOCR-related suppression remains within 50 ms after the end of the noise [36], and we can separate the MOCR-related suppression from suppression due to stimulus acoustic ringing, which generally disappears within a few milliseconds, by setting an adequate interval between the elicitor noise and click. The elicitor noise had a duration of 120 ms, including a 10-ms raised-cosine ramp. The elicitor noise and click were presented alternately with 5- and 75-ms silent gaps between the end of the elicitor noise and the click and between the click and the onset of the next elicitor noise, respectively. One measurement block was composed of the without-noise and with-ipsilateral-noise conditions. In the without-noise condition, the click was presented 50 times at intervals of 30 ms without the elicitor noise. In the with-ipsilateral-noise condition, the combination of elicitor noise and click were presented 50 times. In each measurement block, the without-noise condition was always presented before the with-ipsilateral-noise condition.

One set of MOCR measurements was composed of two blocks of contralateral-MOCR measurement and ten blocks of ipsilateral MOCR measurement. The order of the blocks in one measurement set was randomized. This MOCR measurement set was repeated three times for both ears alternately (the order of left- and right-ear MOCR measurements was randomized for each participant). Thus, a total of six and 30 blocks of contralateral and ipsilateral MOCR measurements, respectively, were performed. Accordingly, for a given ear, each participant was presented with a total of 1800 clicks without noise, 1500 clicks with the contralateral noise, and 1500 clicks with the ipsilateral noise.

Recorded waveforms were averaged for the without-noise, with-ipsilateral-noise, and with-contralateral-noise conditions. MOCR was computed by using the level of CEOAEs that were band-pass filtered between 1 and 3 kHz, in which the largest MOCR-related CEOAE suppression was observed [37]. The pressure levels of the band-pass filtered CEOAEs for each condition were defined as RMS values in the region of 8–18 ms and are denoted as P_withc_, P_withi_ and P_without_ (in Pa). Contralateral and ipsilateral MOCR strength was defined as 20log_10_(P_withc_/P_without_) and 20log_10_(P_withi_ /P_without_), respectively. To specify the frequency region where noise exposure has the largest effect, CEOAEs for the without-noise condition were analyzed in three frequency bands centered at 1, 2, and 4 kHz using a one-octave-wide band pass filter. The pressure level at each frequency (in dB SPL) was defined as an RMS value in the 8–18 ms region of the band-passed waveform. In the following, we refer to those CEOAEs in the without-noise condition as “CEOAE”.

### Time schedule of the measurements

Audiograms, CEOAEs, and MOCR strength were obtained just before and immediately after the one-hour instrument practice session. To observe the recovery from the temporary deterioration, we also conducted the same measurement 60 minutes after the end of the violin practice. The order of hearing thresholds and CEOEAs and MOCR strength measurement was randomized for each participant. For a given participant, the order was identical for the pre- and post-exposure measurements. The measurement of hearing thresholds took approximately five minutes; that of CEOAEs and MOCR strength took approximately 15 minutes.

### Exposure assessment

Participants were instructed to play the violin as in their usual solitary practice. Throughout the instrument practice session, participants played the violin almost continuously, except for short breaks to prepare scores for a next piece. The sounds to which they were exposed during the one-hour practice were recorded by small microphones placed 1–2 cm away from either ear. The average exposure level throughout the one-hour of violin playing was computed with A-weighted root-mean-square (in dBA), 1/3 octave band and octave band sound levels (in dB SPL) with center frequencies between 250 and 10000 Hz.

### Statistical analysis

The effect of the exposure during the practice on CEOAE and audiogram was assessed by a two-way split-plot analysis of variance (ANOVA) with condition (before and after the practice) and frequency as factors. Subsequent post-hoc pairwise comparisons were performed to separately test significance of differences between the data before- and immediately after the practice and between the data before and 1h after the practice. Relationships among changes in CEOAE or audiogram with exposure level and the MOCR strength were assessed by using Pearson’s product-moment correlation coefficient. For all analyses, normality of distribution and equality of variance were ascertained by a one-sample Kolmogorov—Smirnov test and Levene’s tests, respectively. Statistical significance was set at a probability value of p = 0.05 except for the post-hoc pairwise comparisons in which p-values were adjusted by Bonferroni correction.

## Results

### Characteristics of exposure

**Fig 1:**
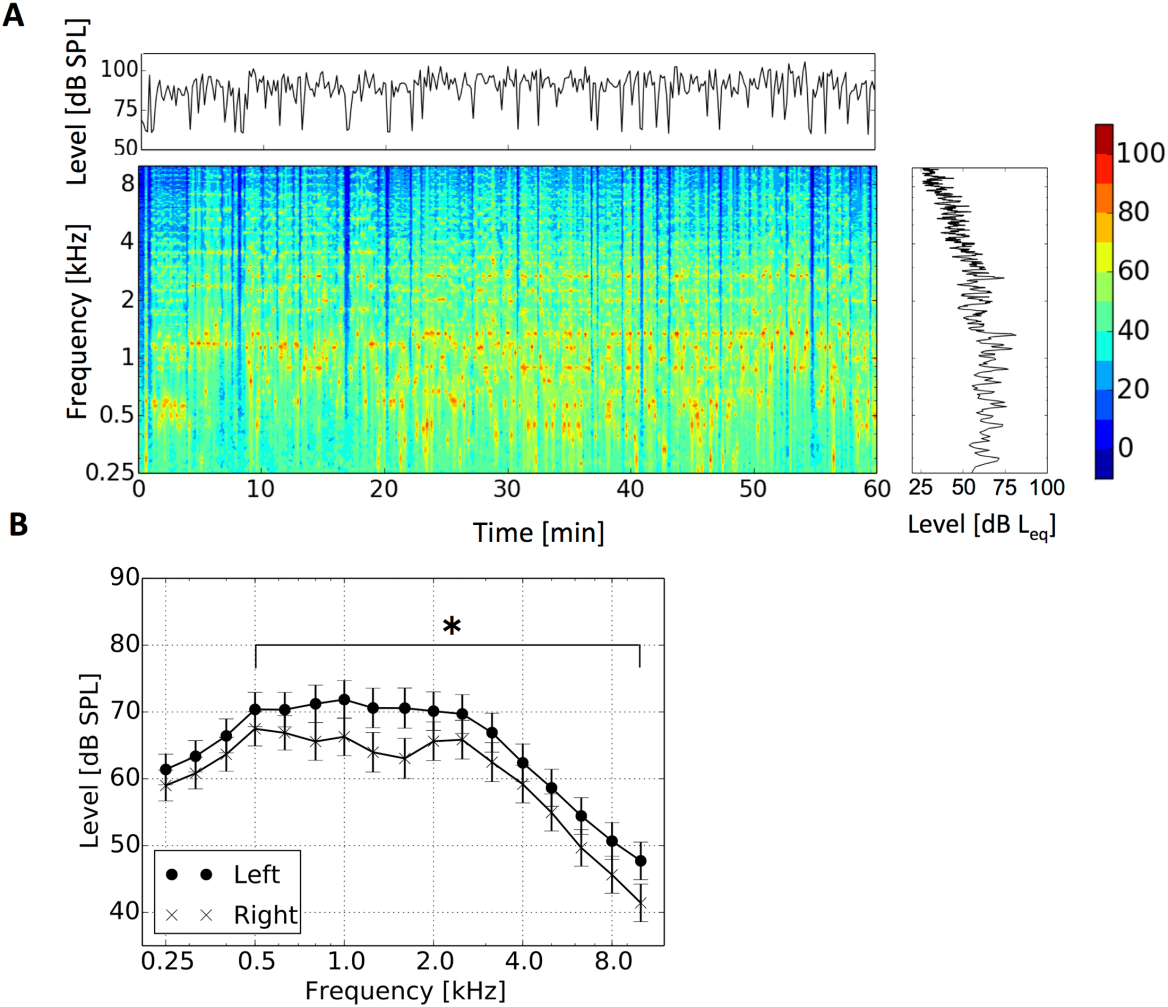
Acoustic characteristics during the violin practice. A: Typical characteristics of the acoustic exposure during a violin practice for the left ear. B: Comparison between exposure level for the left ear and right ear (* *p*<0.0029, Bonferroni-corrected α).

During the violin practice, participants played the violin almost continuously (Fig 1A). A-weighted exposure levels to the right and left ear were 89.7 (SD 2.4) and 98.9dBA (SD 5.9), respectively. The exposure level to the left ear was 9.2 dB (SD 4.3) larger than to the right ear (*T*_15_ = 8.4, *p*<0.001). The exposure level to the left ear was larger than to the right ear in the middle- to high-frequency region (> 500 Hz) (Fig 1B): A two-way split-plot analysis of variance (ANOVA) with laterality (left and right) and frequency as factors showed a significant main effect (*F*_1, 15_ = 30.0, *p*<0.001) and significant interactions of laterality and frequency (*F*_16, 240_ = 10.3, *p<0*.*001*). Significant simple main effects were found at frequencies above 500 Hz (*F*_1,255_> = 9.7, *p*< = 0.0021, Bonferroni-corrected α = 0.0029).

### General characteristics of the measure before the instrument practice

All participants showed normal audiograms (HL < = 25 dB HL) before the violin practice and no significant difference between the right and left ear: A two-way split-plot ANOVA with laterality and frequency as factors shows no significant main effect (*F*_1, 15_ = 0.43, *p* = 0.52) or interaction of laterality and frequency (*F*_4, 60_ = 1.50, *p* = 0.21). There was also no significant difference in CEOAE between the right and left ear (ANOVA as above, *F*_1, 12_ = 0.47, *p* = 0.5 and *F*_2, 24_ = 0.81, *p* = 0.46, respectively).

CEOAE level was significantly smaller in the with-contralateral-noise and with-ipsilateral-noise condition than the without-noise condition both for the right (*T*_12_ = 5.7, *p*<0.001 and *T*_12_ = 4.0, *p* = 0.0017, respectively) and left ear (*T*_12_ = 5.3, *p*<0.001 and *T*_12_ = 4.2, *p* = 0.0012, respectively). Mean ipsilateral MOCRs were -2.0 dB (SD 1.7) for the left ear and -2.2.dB (SD 1.9) for the right ear. Mean contralateral MOCRs were -2.5 dB (SD 1.6) for the left ear and -2.6 dB (SD 1.6) for the right ear. There was no significant difference between the right and left ear for the contralateral MOCR (*T*_12_ = 0.25, *p* = 0.81) and ipsilateral MOCR (*T*_12_ = 0.43, *p* = 0.68).

### Audiogram and CEOAE changes caused by the short-duration instrument practice

**Fig 2:**
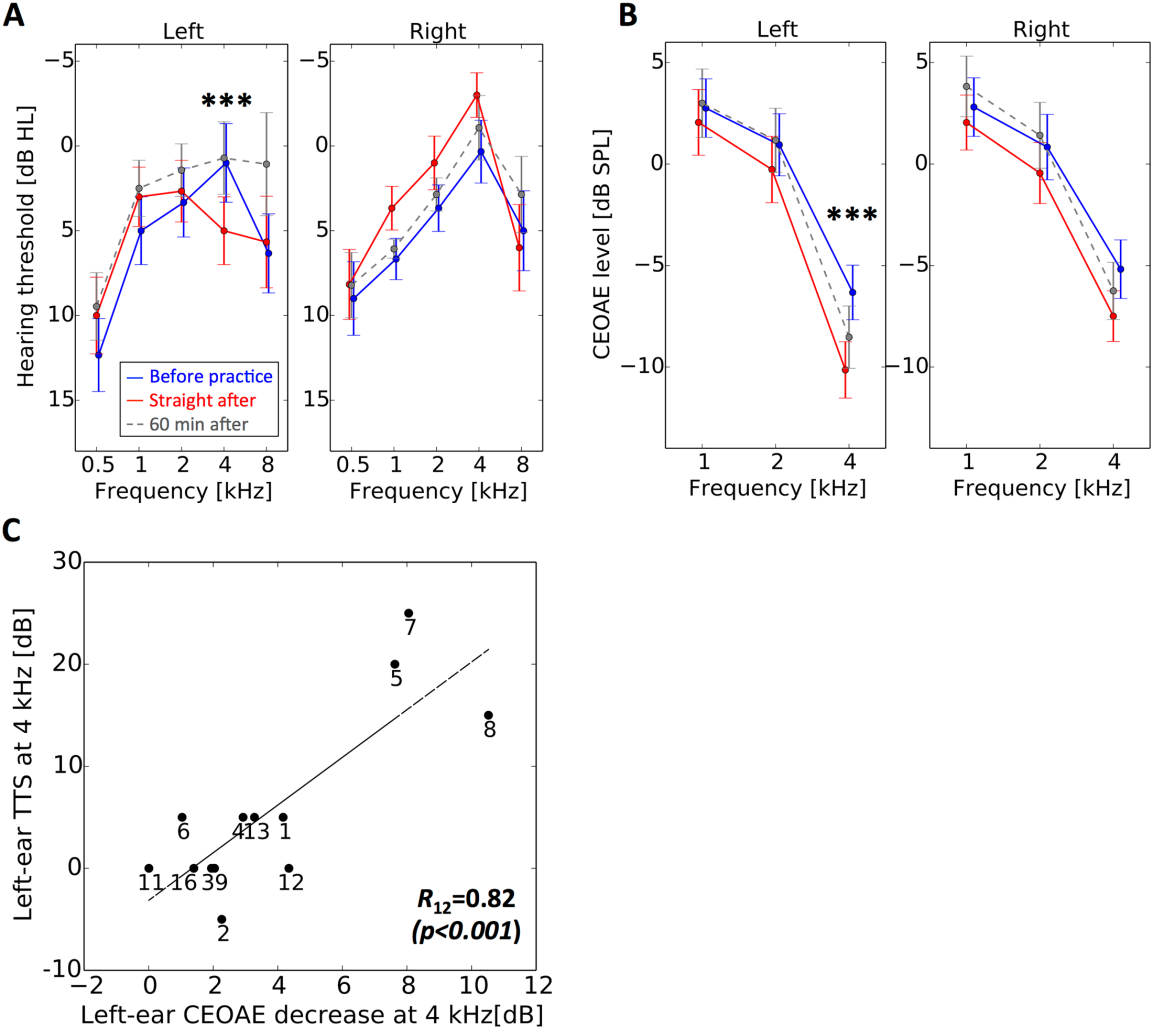
Transient hearing deterioration caused by the short-duration violin practice. A: The audiogram of left ear (left panel) and right ear (right panel) before (blue line) and after (red line) the violin practice. B: The CEOAE spectrum of left ear (left panel) and right ear (right panel) before (blue line) and after (red line) the violin practice. Error bars show the standard errors across listeners. Significance of post-hoc pairwise comparisons is marked as *** *p*<0.001 and * *p*<0.05. The data obtained 60 minutes after the end of the violin practice are shown by gray dashed lines. **C**: Correlation between CEOAE decrease at 4 kHz and TTS at 4 kHz. Each participant’s data point is represented with participant number.

The short-duration instrument practice caused a left-ear and 4-kHz specific TTS (Fig 2A): For left ears, a two-way split-plot ANOVA with condition (before and after the practice) and frequency as factors showed no significant main effect (*F*_1, 15_ = 0.0021, *p* = 0.96) and a significant interaction of condition and frequency (*F*_4, 60_ = 4.2, *p* = 0.0048). A simple main effect was observed at 4 kHz in audiograms (*F*_1, 75_ = 7.9, *p* = 0.0063, Bonferroni-corrected α = 0.01). An ANOVA as above for the right ear showed no significant main effect (*F*_1, 15_ = 0.52, *p* = 0.48) or interaction (*F*_4, 60_ = 1.1, *p = 0*.*38*). The right-ear hearing thresholds straight after the practice were lower in 1–2 kHz than before practice, but those differences were not statistically significant.

The short-duration instrument practice caused a 4-kHz specific CEOAE decrease in both ears (Fig 2B): For left ears, a two-way split-plot ANOVA with condition (before and after the practice) and frequency as factors showed a significant main effect (*F*_1, 12_ = 20.4, *p*<0.001) and a significant interaction of condition and frequency (*F*_2, 24_ = 15.1, *p*<0.001). An ANOVA as above for the right ear showed no significant main effect (*F*_1, 12_ = 4.1, *p* = 0.067) or a significant interaction (*F*_2, 24_ = 3.1, *p* = 0.062). A simple main effect was observed at 4 kHz in the left and right ear (*F*_1,36_ = 48.1, *p*<0.001, Bonferroni-corrected α = 0.017 and *F*_1,36_ = 8.2, *p* = 0.0069, Bonferroni-corrected α = 0.017, respectively). The TTS at 4 kHz and the CEOAE decrease at 4 kHz were significantly correlated (*R*_12_ = 0.82, *p*<0.001; Fig 2C).

### Correlation between exposure level and temporary hearing deterioration

**Fig 3:**
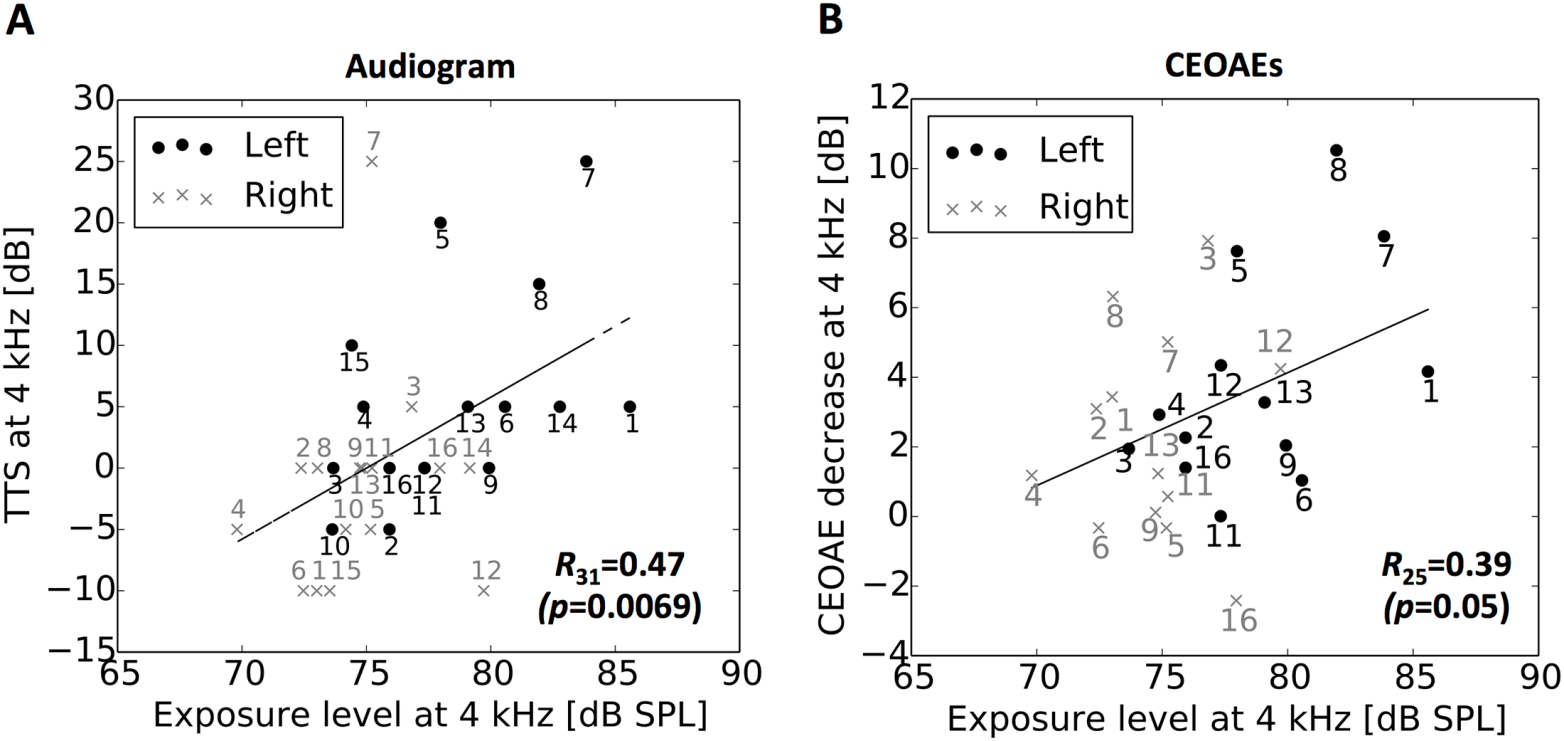
Amount of hearing deterioration reflects exposure level. Correlation of exposure level at 4 kHz with the elevation of hearing threshold at 4 kHz (A) and with CEOAE decrease at 4 kHz (B). The data for the left and right ears are represented by the dots and crosses, respectively. The numbers attached to the symbols are participant numbers. The Pearson correlation coefficients, R, with their p-values shown in the panels, and the regression lines were derived for the pooled data for both ears.

The larger TTS and the greater CEOAE decrease in the left ear are likely to reflect the larger exposure level in the left ear than in the right ear (Fig 3A and 3B): When the data for the left and right ear were pooled, the one-octave exposure level at 4 kHz was significantly correlated with the TTS at 4 kHz (*R*_31_ = 0.47, *p* = 0.0069) and the CEOAE decrease at 4 kHz (*R*_25_ = 0.39, *p* = 0.050); the data for the left ear were distributed around the right top, and the data for the right ear were distributed around the left bottom on the regression line derived from the exposure level and the size of hearing deterioration (Fig 3A and 3B). Nevertheless, separate analyses showed a tendency towards (although not significant) positive correlation of exposure level with TTS (*R*_15_ = 0.48, *p* = 0.057 for left ear; *R*_15_ = 0.24, *p* = 0.37 for right ear) and CEOAE decrease (*R*_12_ = 0.49, *p* = 0.092 for left ear; *R*_12_ = 0.12, *p* = 0.69 for right ear) only for the left ear.

### Correlation between MOCR strength and temporary hearing deterioration

**Fig 4:**
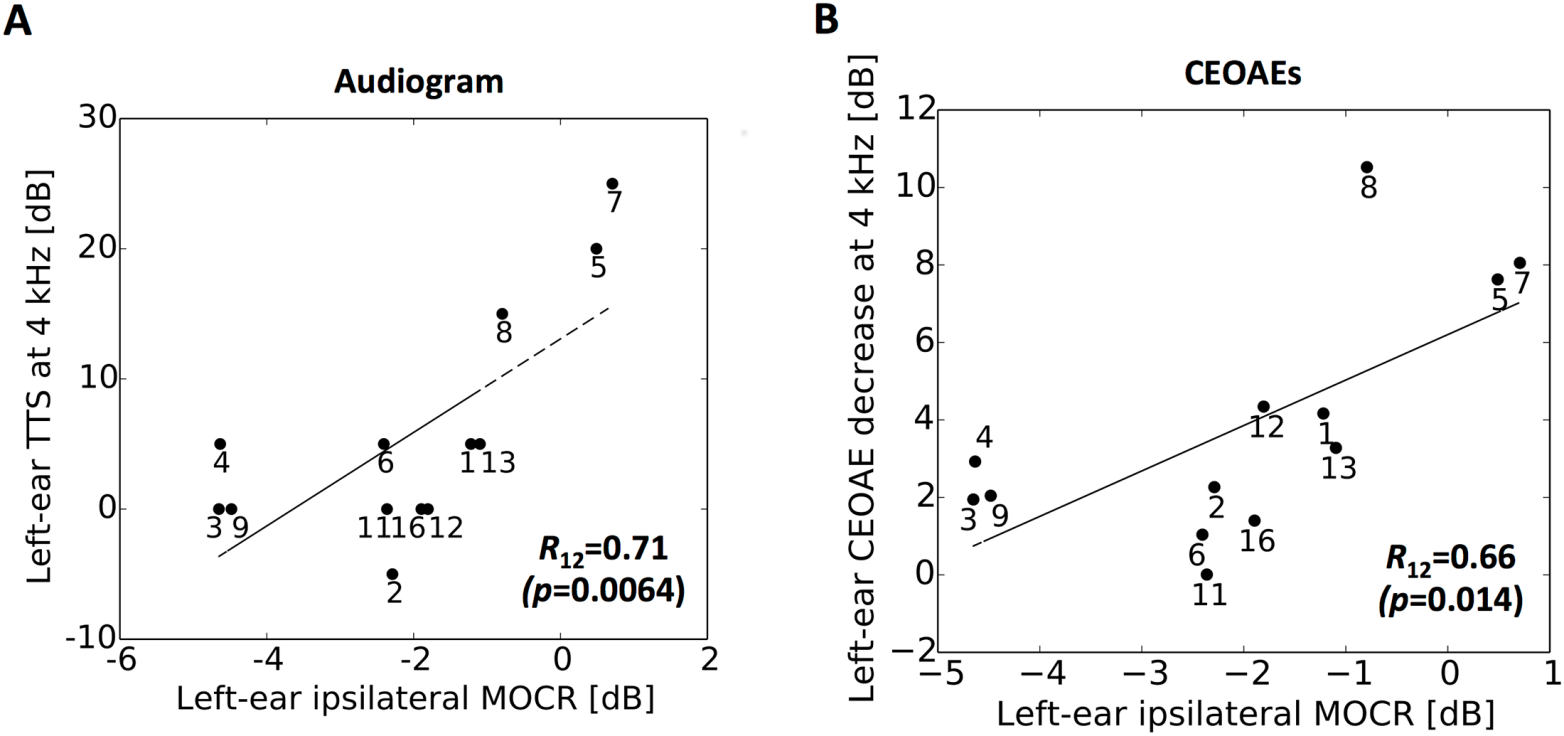
MOCR strength could predict the size of hearing deterioration caused by a violin practice. Correlation of ipsilateral MOCR strength with the elevation of hearing threshold at 4 kHz (A) and with CEOAE decrease at 4 kHz in the left ear (B). Pearson correlation coefficients are shown with their p-value in parentheses. Regression lines were derived from linear least squares regression. Each participant’s data point is represented with participant number.

For the left ear, ipsilateral MOCR strength was negatively correlated with the TTS (*R*_12_ = 0.71, *p* = 0.0064; Fig 4A) and the CEOAE decrease (*R*_12_ = 0.66, *p* = 0.014; Fig 4B). For the right ear, MOCR strength was not correlated with the hearing threshold changes and CEOAE decrease (*R*_12_ = 0.21, *p* = 0.49 for audiogram; *R*_12_ = 0.18, *p* = 0.56 for CEOAE). Contralateral MOCR strength was not significantly correlated with the TTS or the CEOAE decrease in the right and left ear (|*R*_12_|<0.27, *p*>0.37).

### Recovery from temporary hearing deterioration

To observe the recovery from the temporary deterioration, we also conducted the same measurement 60 minutes after the end of the violin practice and found that the left-ear hearing thresholds were lower (better audibility) than before the practice (Fig 2A): A two-way split-plot ANOVA with condition (before and 60 minutes after the practice) and frequency as factors showed a significant main effect (*F*_*1*, *13*_ = 7.4, *p* = 0.018) and no significant interaction of condition and frequency (*F*_*4*, *52*_ = 1.7, *p* = 0.16) in the left ear. An ANOVA as above showed no significant main effect or interaction in the right ear (*F*_*1*, *13*_ = 1.3, *p* = 0.27 and *F*_*4*, *52*_ = 0.31, *p* = 0.87 respectively).

On the other hand, a significant CEOAE decrease persisted even 60 minutes after the practice for the left ear (Fig 2B): An ANOVA as above showed no significant main effect *(F*_1, 11_ = 3.83, *p* = 0.076) and a significant interaction (*F*_*2*, *22*_ = 8.25, *p* = 0.0021). The simple main effect at 4 kHz was significant (*F*_*1*, *33*_ = 15.9, *p*<0.001, Bonferroni-corrected α = 0.017). For the right ear, there was no significant difference before and 60 min after the practice: An ANOVA as above showed no significant main effect *(F*_1, 11_ = 0.0041, *p* = 0.95) or significant interaction (*F*_*2*, *22*_ = 5.90, *p* = 0.0089). There was no simple main effect (*F*_*1*, *33*_<4.65, *p*>0.039, Bonferroni-corrected α = 0.017).

## Discussion

In this study, we showed that MOCR strength could predict hearing deterioration caused by a short-duration instrument practice. To our knowledge, this is the first study to report an association between MOCR strength and the risk of hearing loss among musicians.

### General characteristics of the measures

There was no significant difference between the left and right ear for audiograms and CEOAE before the violin practice. In contrast, some studies have reported that professional violinists tend to have left-ear-specific hearing loss in audiograms [3], [6]. Presumably, our participants were younger than those in previous studies [3], [6] and had not developed permanent hearing loss in the left ear.

Although we should be cautious when comparing our results with previous studies because of several methodological differences [38], [31], we note an interesting difference from those previous studies. Our participants showed no significant left-and-right-ear difference in the MOCR strength. Generally, the MOCR of the right ear has been reported to be stronger than that of the left ear for both musicians and non-musicians [38]. This inconsistency might be due to the asymmetry of exposure in violinists (i.e., larger exposure in the left ear than in the right ear). An experimental animal study has shown that repeated exposures induce a functional increase in the activity of MOC fibers [39]; therefore, the larger exposure to the left ear might make the left-ear MOCR stronger and comparable to the right-ear MOCR.

### Temporary hearing deterioration revealed in audiogram and CEOAE

An instrument practice as short as one-hour caused a 4-kHz and left-ear specific TTS, which is similar to a typical pattern of the PTS among professional violinists [4]. Consistently, CEOAE for the left ear decreased at 4 kHz proportionally to the TTS. This left-ear specific TTS and CEOAE decrease could be accounted for by larger exposure to the left ear than to the right ear, related to the proximity of instruments to the ears [3], [5], [6]. This is consistent with the positive correlation we found between the left-ear TTS and exposure level, although the p-value did not reach the criterion value for the statistical significance, probably because of insufficient statistical power.

CEOAE decreased at 4 kHz also for right ears. CEOAE might be sensitive to a slight functional change of the right ear that could not be revealed in the conventional audiometry. It has been shown that CEOAE can exhibit an observable change before conventional audiometry changes [33].

### Recovery from temporary hearing deterioration

Interestingly, the left-ear hearing thresholds one hour after a violin practice were even lower than those before it. Considering that a significant CEOAE decrease persisted even 60 minutes after the practice, this enhanced hearing sensitivity may originate in functional changes in the retrocochlear auditory pathway. Indeed, several animal studies observed enhancement of neural responses after acoustic exposure: Salvi et al. [40] measured evoked responses in inferior colliculus before and after exposure to a 2 kHz pure tone of 105-dB SPL and observed larger maximum responses at 0.5 and 2 kHz after the acoustic exposures. Willott and Lu [41] reported that some neurons showed enhanced evoked responses immediately after the exposure to 95- or 100-dB white noise. Taken together, the acoustic exposure during the violin playing might induce short-term plastic changes in the central auditory pathway, leading to the enhanced hearing sensitivity after a practice.

### Prediction of individual susceptibility to noise exposure by MOCR measurement

MOCR strength was negatively correlated with the TTS and the CEOAE decrease caused by a short-duration instrument practice; the participants who had strong efferent activity tended to show a small TTS and small OAE decrease. This result is consistent with studies of NIHL reporting that individuals with a stronger MOCR show a smaller TTS [23] (in humans) and smaller PTS [15] (in guinea pigs) induced by intense white noise.

Despite extensive studies [22], the evidence for the protective role of MOCR in *humans* is still equivocal. Especially under field study conditions in which noise exposures are varied to some extent among participants, no earlier study reported that MOCR strength can predict hearing deterioration: MOCR strength did not correlate with the TTS caused by exposure to one day of occupational noise [30], three hours of a discotheque [29], five gun shots [27], or one hour of music from an MP3 player [28]. An exception was that a significant correlation was found between MOCR strength and threshold *recovery* from TTS caused by exposure to rifle discharge [26]. The critical difference between those previous studies and the current one was that our participants were musicians. The relation between the MOCR strength and hearing deterioration might be clearer in the current study than those in previous ones; Musicians’ MOCRs are stronger than those of non-musicians [31], [32], [38]. They therefore would generate a larger variation, which could be a dominant factor determining the hearing deterioration. Unfortunately, we cannot make a direct comparison of the variability with those previous studies because of several methodological differences (described below); however there is some indirect evidence: In Perrot et al. [38], the variability of the contralateral MOCR in musicians was larger than in non-musicians (its statistical significance was not mentioned), whereas both the variability and average of the ipsilateral MOCR strength in the current study were larger than those in non-musicians reported in Berlin et al. [36].

Regarding differences between our study and previous ones [26], [28], [29], [30], it should be noted that the temporary hearing deterioration was correlated with the ipsilateral MOCR but not with the contralateral MOCR in our experiments. This is consistent with the fact that previous studies have failed to find a correlation between contralateral MOCR and TTS [26], [28], [29], [30]. However, it is still unclear whether the ipsilateral MOCR plays a more important role in protection against NIHL than the contralateral MOCR; During the violin practice, the ipsilateral MOCR to the left ear was more activated by stronger exposure to the violin sound than the contralateral MOCR, which was activated by the exposure to the right ear. As a result, the ipsilateral MOCR might provide stronger noise-protective role and more predominantly determine the size of the temporary hearing deterioration than the contralateral MOCR.

It is not yet clear why musicians’ MOCR (or its variation) is larger than non-musicians’ and what produces the inter-individual variation of MOCR strength among musicians. Perrot and Collet [32] suggested that there are two possible neuroplastic factors that may enhance the musicians’ MOC system activity: sound conditioning of the MOCR due to the exposure to musical sounds and central nervous plasticity caused by active musical practice. As to sound conditioning, it is known that sound exposure itself induces plastic changes in the MOC system. Brown et al. [39] reported that repeated sound exposure induces a functional increase of the activity of MOC fibers, which might reflect a long-term neuroplasticity in the SOC [42]. Kujawa and Liberman [43] have also shown that the PTS induced by intense sound is smaller after long-term exposure to a moderate level of sound and that the smaller PTS is accompanied by an enhancement of MOCR. Active training during musical practice can also vary the MOCR strength. Boer and Thornton [44] reported that training for a speech-in-noise discrimination task improved the task performance, concomitantly with an increase in MOCR strength. This improvement presumably reflects training-induced short-term plasticity of corticofugal neural pathways, as demonstrated in experimental animals [45], [46]. An alternative to the possible involvement of neuroplasticity in developing a strong MOCR would be the notion that people whose MOCR is innately stronger are more likely to become musicians. In other words, the MOCR might constitute innate predispositions to music, e.g., MOCR might facilitate musical training, and people with innately stronger MOCR would show greater musicianship. Taken together, the differences in exposure and active training during musical practice as well as innate biological factors might produce the inter-individual difference in musicians’ MOCR strength.

## Conclusion

A short-duration instrument practice causes a temporary hearing deterioration among violinists. The larger exposure level in the left ear could account for the more pronounced hearing deterioration in the left ear than in the right ear, but it could not entirely explain the inter-individual variation of the hearing deterioration. On the other hand, MOCR strength assessed by CEOAE suppression could predict the size of the hearing deterioration moderately well. Our findings imply that the exposure level depending on the proximity of the instrument can partly determine the risk of hearing loss and that MOCR measurement is promising for assessing the risk of hearing loss among musicians.
